# Lignocellulosic Biomasses from Agricultural Wastes Improved the Quality and Physicochemical Properties of Frying Oils

**DOI:** 10.3390/foods11193149

**Published:** 2022-10-10

**Authors:** Eman Ahmed, Ashraf Zeitoun, Gamal Hamad, Mohamed A. M. Zeitoun, Ahmed Taha, Sameh A. Korma, Tuba Esatbeyoglu

**Affiliations:** 1 Department of Food Science, Faculty of Agriculture (Saba Basha), Alexandria University, Alexandria 21531, Egypt; 2 Department of Food Technology, Arid Lands Cultivation Research Institute (ALCRI), City of Scientific Research and Technological Applications (SRTA City), New Borg El-Arab 21934, Egypt; 3 Department of Functional Materials and Electronics, Center for Physical Sciences and Technology, Saulėtekio al. 3, 10257 Vilnius, Lithuania; 4 Department of Food Science, Faculty of Agriculture, Zagazig University, Zagazig 44519, Egypt; 5 School of Food Science and Engineering, South China University of Technology, Guangzhou 510641, China; 6 Department of Food Development and Food Quality, Institute of Food Science and Human Nutrition, Gottfried Wilhelm Leibniz University Hannover, Am Kleinen Felde 30, 30167 Hannover, Germany

**Keywords:** fried oil, adsorbent, lignocellulosic biomass, fatty acid, food waste, oil regeneration, oil quality, sustainability

## Abstract

In this work, the effects of using natural lignocellulosic-based adsorbents from sugarcane bagasse (SC), cornstalk piths (CP), and corn cob (CC) on the physicochemical properties and quality of fried oils were studied. The properties of lignocellulosic biomasses were examined using X-ray diffraction (XRD), scanning electron microscope (SEM), and Fourier transform infrared spectroscopy (FTIR). Moreover, the changes in the physicochemical properties of fresh, fried oils (for 4, 8, 12, 16 and 20 h) and adsorbents-treated oils were examined. The XRD results revealed that SC and CP biomasses have more amorphous regions than CC biomass, which had the highest crystallinity percentage. The results also showed that lignocellulosic biomasses enhanced the quality of the used oils. SC was the most effective biomass to enhance the properties of the used sunflower oil. For instance, the acid value of oil samples fried for 20 h reduced from 0.63 ± 0.02 to 0.51 ± 0.02 mg KOH/g oil after SC biomass treatment. For the peroxide value, the SC biomass treatment reduced it from 9.45 ± 0.56 (fried oil for 20 h) to 6.91 ± 0.12 meq O_2_/kg. Similarly, SC biomass adsorbent reduced the *p*-Anisidine Value (*p*-AV) of the used oil (20 h) from 98.45 ± 6.31 to 77.92 ± 3.65. Moreover, SC adsorbents slightly improved the lightness of the used oils (20 h). In conclusion, natural lignocellulosic biomasses, particularly SC, could be utilized as natural adsorbents to improve the oil quality. The results obtained from this study could help in developing sustainable methods to regenerate used oils using natural and cheap adsorbents.

## 1. Introduction

Deep-fat frying is an extensively used cooking approach worldwide as fried products have a unique texture, color and flavor [1]. In this cooking approach, the food product is immersed in edible oil at temperatures ranging from 120–190 °C [2]. Frying also involves simultaneous mass and heat transfer in both directions, from the frying medium “edible oil” to the food product and vice versa. For example, in the case of French fries, the oil penetrates potato strips while a few soluble materials, water and starch, escape from the strips [3,4]. The physicochemical changes of the frying medium and the fried product are influenced by several process factors, including frying time, frying oil temperature and nature of the fried products (i.e., thermal characteristics and moisture content) [4]. The presence of oxygen with high temperatures and moisture can cause oil degradation and decrease the quality of the fried products. Oil degradation due to oxidation [5], hydrolysis [6], and polymerization [7] can form undesirable components such as free fatty acids, dimers, cyclic compounds, alcohols, polymers and acrylamides (a chemical carcinogen formed via the Maillard reaction between reducing sugars and asparagine amino acid) [8,9]. The consumption of a diet produced by repeatedly heated oil could increase the risk of severe health issues, including cardiovascular diseases [10], cancers [11], impaired renal function [12], hepatorenal toxicity [13], hypertension and atherosclerosis [14].

Recent studies estimated that the generation of the used frying oils is about 20–32% of the total vegetable oils consumption. Thus, the global cooking oil production is around 42 million tons per year [15]. Discarding the used oils within solid residues or through siphons and sinks causes many environmental and economic issues. This could increase the maintenance costs of waste treatment plants and sewage, encourage the proliferation of pests and vectors, trigger pollution and public health issues and damage the infrastructure. Moreover, discarding the used oils to soil resources can damage the ecosystem as oil isolates soil from water and air, killing useful natural organisms such as bacteria and worms. In the water resources, waste oil pollution could block the sunlight and reduce the oxygen content, damaging aquatic fauna and flora [15,16]. Thus, the reutilization of waste frying oils is highly recommended to mitigate the mentioned negative effects.

Passive and active filtrations have been used to regenerate used oil. Passive filtration via filter paper and cloth only removed coarse particles; however, fine particles and water remained in the oil. In comparison, active filtration via adsorptive properties of natural and synthetic adsorbents removes chemical substances, including free fatty acids, peroxides, dimers, dark color matter and aldehydes [9,17]. There are many advantages of adsorption-based filtration of the used oil, such as low generation of wastewater, low energy consumption and suitability for any quality of oil [15]. On the other hand, the main drawbacks of active filtration are the high consumption of adsorbents, the unavailability of effective filtration equipment and the leaching of some metals into oils [18]. Several types of natural (i.e., clays, ceramics, etc.) and chemical or synthetic materials (bentonite, calcium silicate, magnesium oxide and aluminum hydroxides) can be used for active filtration of the used cooking oils [19]. There is a rising interest in natural and sustainable materials due to the higher costs and toxicity concerns of synthetic adsorbents. Thus, raw materials from agricultural wastes could be a suitable alternative to conventional chemical adsorbents thanks to their low cost, availability and biodegradability [9,20]. The lignocellulosic biomass from agricultural wastes, including sugarcane bagasse, corn cobs, rice straw, corn piths, orange peels and wheat bran, can be utilized as natural adsorbents because of their extensive production and availability [21,22,23,24].

Corn cobs (CC) are solid residues from corn production; they contain lignin (20.3%), hemicellulose (48.7%), cellulose (~32%) and acetyl groups (3.40%) [25,26]. Several studies used CC as an effective adsorbent in different applications. For example, Alves et al. [27] used modified CC to adsorb phenylalanine and tyrosine from an aqueous solution. Bavaresco et al. [24] evaluated the potential of using CC to reduce the acidity of the used oils to produce biodiesel. Cornstalk piths (CP) are found in the center of cornstalk, forming approximately 50% of cornstalk by weight. CP comprises around 37% cellulose, 13% lignin and 24% pentosane and contains ash and other traces [28]. CP was applied as an affordable adsorbent during wastewater treatment to remove dyes, spilled oil and toxic metal [29,30,31].

Moreover, malic acid-modified CP was successfully used to adsorb methylene blue and crystal violet dyes [31]. Sugarcane bagasse piths (SC) is the byproduct of biomass residue after extracting juice from sugarcane, and it is mainly used in sugar industry factories to run boilers [32]. SC consists of around 40% cellulose and almost 32% of hemicellulose, and 5% lignin and other traces [33]. Wannahari et al. [34] utilized SC to recover the used palm oil; 5 *w*/*v* of bagasse caused a reduction in the free fatty acids to 82.14% and reduced the color density to 75.67%.

Therefore, we hypothesized that lignocellulosic materials could restore some original attributes of the used frying oils because of their morphological properties, such as the spores at the surface of their particles. Moreover, these materials could have functional groups that act as active sites to adsorb some chemical residues resulting from oil deterioration. To date, few studies investigated the potential of using natural lignocellulosic biomass to enhance the quality of the used frying oils [9,35,36].

The effects of using CC, CP and SC as natural lignocellulosic-based adsorbents on the quality of the used sunflower oils are yet to be studied. Thus, this work aims to evaluate the effects of adding using some lignocellulosic biomass materials (CC, CP and SC) on the quality of sunflower used oil. Standard sunflower oil mainly consisted of linoleic acid (C18:2, 48.3–74.0%), oleic acid (C18:1, 14.0–39.4), palmitic acid (C16:0, 5–7.6%) and stearic acid (C:18:0, 2.7–6.5%) [37].

In this work, the physicochemical and morphological properties of the lignocellulosic materials were investigated using XRD, FTIR and SEM. Lignocellulosic biomass mainly comprised lignin (10–25%), hemicellulose (20–35%), and cellulose (35–50%) with traces of ash and extractives [38]. The FTIR, SEM and XRD techniques are usually used to investigate the physicochemical and microstructural properties of lignocellulosic biomasses [39]. The results obtained from these techniques could help to interpret the physicochemical properties of the used frying oils before and after treatment with adsorbents. Furthermore, the physicochemical properties of the fried sunflower oil were examined after using the lignocellulosic materials as natural adsorbents.

## 2. Materials and Methods

### 2.1. Materials

The corn cobs (CC) and cornstalk piths (CP) from corn stover (*Zea maize*, cultivar 30 K 8, produced by Corteva Agriscience LLC., New Cairo, Egypt) were collected from a local corn farm in Beheira governorate, Egypt. Sugarcane bagasse (SC) wastes were collected from a local sugarcane juice producer immediately after squeezing for juice production. Sunflower oil (Crystal brand from Arma for the food industry, Al-Sharqia Governorate, Egypt) was purchased from a local market in Alexandria, Egypt. Frozen potato strips (10 × 10 × 50 mm) were bought from Farm Frites (Tenth of Ramadan City, Egypt). Ethanol, NaOH, KOH, phenolphthalein, potassium iodide, glacial acetic acid, chloroform, sodium thiosulfate and starch were purchased from Elnasr chemical company and Elgomhouria pharmaceutical company (Cairo, Egypt). Isooctane was purchased from the Alfa chemical group (Cairo, Egypt). *n*-Hexane, sodium hydrogen sulfate, *p*-AV and methyl esters fatty acid standards were obtained from Sigma Chemical Co., Ltd. (St. Louis, MO, USA). All reagents used in this investigation were of analytical grade.

### 2.2. Methods

#### 2.2.1. Adsorbents Preparation

The preparation of lignocellulosic adsorbents (SC, CP and CC) was performed once, according to Schneider et al. [35], with minor modifications. The corn wastes (CC and CP) were collected a month after cultivation directly from the corn farm. After collection, SC wastes were dried in a sunny area for 24 h (daytime only). Then, all biomasses were kept in a closed package in the refrigerator until further experiments. After that, 500 g of each adsorbent material was washed using distilled water to remove the dust particles and left in a sunny and dry area for two days, then dried in the oven for 24 h at 90 °C. Then, the dried products were ground in a grinder and sieved using sieves of 50 mesh (~0.3 mm). These three types of adsorbents were selected based on preliminary results, which showed that these adsorbents had better performance than other tested absorbents (including orange and banana peel biomasses). The released pigments from orange and banana peel biomasses changed the color of oil samples to an unacceptable color for consumers.

#### 2.2.2. Scanning Electron Microscope (SEM)

An SEM (Tescan Vega 3, Tescan Company, Brno, Czech Republic) at 20 kV was applied to examine the morphological properties. The SEM was combined with Energy Dispersive X-ray spectroscopy (EDX) to investigate the element contents of the adsorbents [40]. A high vacuum sputtering instrument (Quoroum150T, UK) was used to sputter samples (at 40 mA sputter current for 120 s) with gold.

#### 2.2.3. Fourier Transform Infrared Spectroscopy (FTIR)

The Fourier transform infrared-attenuated total reflectance (FTIR-ATR) spectroscopy was performed to identify the main functional groups of wastes and to get an overview of their chemical structure. The FTIR transmittance spectra were recorded in the frequency range 4000–400 cm^−1^ with 64 scans and 4 cm^−1^ resolution using a VERTEX 70v FT-IR Spectrometer (Bruker Optics, Ettlingen, Germany) [41]. The FTIR spectra were further analyzed and fitted using OriginPro 2021 software (OriginLab Corporation, Northampton, MA, USA) and PeakFit 4.12 software (SeaSolve Software Inc., San Jose, CA, USA). The Levenberg-Marquardt algorithm was used during the peak analysis to perform non-linear fitting of the peaks. Baseline correction was made using a second derivative method for finding anchor points and detecting the baseline.

#### 2.2.4. X-ray Diffraction (XRD)

The crystallinity behavior of the adsorbents was identified using a PW 1390 Philips X-ray diffractometer with Ni-filtered Cu-Kα radiation (λ = 0.15418 nm) energized at 45 kV. The XRD patterns were recorded in reflection mode from 0° to 80° in a diffraction angle range of 2θ. The crystallinity index (*CrI*%) was calculated based on the method of Karthika et al. [42] and Equation (1). Where (*I am*) is the intensity at 2θ = 18.4° and (*I cr*) is the intensity of the main peak around 2θ = 21.6°.

(1) $$Crystallinity\;index\;\left(CrI\%\right)=\frac{\left(I\;cr-I\;am\right)}{I\;cr\;}\times 100$$

#### 2.2.5. Frying Process

An electric deep fryer (Moulinex, Alençon, France) was used for deep frying. Sunflower oil (2.5 L) was poured into the fryer and heated at 180 °C. Fifteen minutes were required to heat the oil to the frying temperature; one batch (150 g potato strips) was fried for 5 min every hour. The frying process lasted for 5 days (4 h/day). About 600 mL of fried oil was withdrawn (each day). The fried oils were allowed to cool to room temperature [9] and mixed with different adsorbents (150 mL for each adsorbent). About 200 mL of fresh oil was added to the frying oil each day (after 4 h of frying) to replace part of the withdrawn fried oil.

#### 2.2.6. Filtration

An amount of 150 mL of the used oil was mixed with 5% (*w*/*w*) adsorbent + 3% (*w*/*w*, citric acid/adsorbent), then mechanically stirred for 30 min and filtered with cloths then with filter papers (Whatman number 41). After filtration, the used (control) and treated oil samples were kept at −18 °C for the physiochemical analysis. Citric acid, as a metal chelator, was added to prevent the oxidation of oil samples during the filtration and adsorption processes, as lignocellulosic biomasses could release some minerals during the adsorption process [43]. Moreover, previous studies showed that citric acid did not reduce the acid value of fried oils [17,44].

#### 2.2.7. Acid Value (AV)

The AV was calculated following the method of the American Oil Chemists’ Society (AOCS Method Cc 5a-40) [45]. Briefly, 5 g of oil was added to hot, neutral ethyl alcohol in a 25 mL elementary. Then, 0.1 N NaOH was used to titrate the mixture and phenolphthalein was used as an indicator. Equation (2) was used to calculate the AV.

(2) $$\mathrm{Acid}\;\mathrm{value}\;(\mathrm{AV})=\frac{\mathrm{mL}\times N\;\times \;56.1}{\mathrm{sample}\;\mathrm{weight}}$$

where 56.1 = molecular weight of KOH, N = normality of NaOH and mL = milliliter of required NaOH.

#### 2.2.8. Peroxide Value (PV)

The PV was measured based on the method of Nuru and Getachew [46]. Five gram of each sample was poured into a 250 mL Erlenmeyer flask, 0.5 mL of saturated potassium iodide solution, and 30 mL of chloroform-glacial acetic acid (2:3, *v*/*v*) were mixed and kept in a dark place for 1 min at room temperature (~25 °C). Then, distilled water (30 mL) was added to terminate the reaction then saturated starch (2 mL) was added as an indicator. Subsequently, the mixture (with dark brown to purple color) was titrated with standardized 0.12 M sodium thiosulfate solution until the disappearance of the color. The PV was expressed as milliequivalent of oxygen/kilogram of oil (meq O_2_/kg) following Equation (3).

(3) $$\mathrm{Peroxide}\;\mathrm{value}\;(\mathrm{PV})=\frac{(S-B)\;\times \;M\;\times \;1000}{m}$$

where m = sample weight, M = Molarity of Na_2_S_2_O_3_, S = titration of sample and B = titration of the blank.

#### 2.2.9. *p*-AV and the Total Oxidation Value (TOTOX)

The *p*-AV was calculated based on the IUPAC method [47] with a minor modification. Ten milliliters of isooctane were used to dilute a 0.1–0.3 g sample. The adsorption at 350 nm (A_b_) was measured against isooctane as a blank using a UV-VIS spectrophotometer (T80, PG Instruments, Wibtoft, UK). Then, 1 mL of *p*-AV solution was added to 5 mL of fat solution in a tube. The *p*-AV solution (1 mL) was mixed with 5 mL of the solvent in a second tube and shaken. The adsorption was then measured after 10 min (A_s_), and the second tube was used as a blank. The *p*-AV was calculated according to Equation (4). Where m is the mass of the oil sample.

(4) $$\;(p-\mathrm{AV})=\frac{10\;\times \left(1{.2\;\times \;A}_{S}-A_{b}\right)}{m}$$

The TOTOX value was estimated based on the values of *p*-AV and PV following Equation (5) [48].

(5) TOTOX value=2 × PV+p-AV

#### 2.2.10. Fatty Acids Analysis

Fatty acid compositions of fresh, treated and untreated oil samples were determined according to Yang et al. [49] with minor modifications. An amount of 5 mL of *n*-hexane was used to dissolve 100 mg of an oil sample. 11.2 g of KOH was added to 100 mL ethanol (as a transesterification agent) and poured into the mixture. The tube was vortexed for 1 min, and solid sodium hydrogen sulfate (0.5 g) was added to the mixture. The mixture was then centrifuged at 4000× *g* at 25 °C for 3 min then the supernatant was injected for analysis. The obtained methyl esters were examined using an Agilent Technologies 7890A GC (LTD Corporation, Mundelein, IL, USA) combined with a flame ionization detector and equipped with a fused silica capillary column (0.4 mm diameter; 0.25 μm film thickness; 30 m length). The analysis was operated at 0.5 bar pressure, a nitrogen flow rate of 3 mL min^−1^ and an oven temperature of 195 °C. The column and detector temperatures were set at 230 and 250 °C, respectively. The fatty acid methyl esters were identified based on retention time using commercial methyl esters fatty acid standards (Sigma Chemical Co., St. Louis, MO, USA). The peaks were identified using Agilent Technologies ChemStation software.

#### 2.2.11. Color

The color was measured according to the method of Caiping Su and Pamela White [50] using the Hunter Lab instrument (Easy Match QC, Hunter Associates Laboratory, Inc., Reston, VA, USA). For the *L* value, the value closer to 0 reflect black (darkness), and the value closer to 100 reflects whiter (lighter). While a* values indicate green (−*a*) to red (+*a*) colors, and b* represent blue (−*b*) to yellow (+*b*) colors.

#### 2.2.12. Statistical Analysis

Most experiments were performed in triplicates, while the color and fatty acids analysis were performed in duplicates. The data are displayed as means and standard deviations. Data analysis was completed using SPSS version 25 (IBM SPSS Statistics^®^, Chicago, IL, USA). One-way ANOVA and Duncan’s tests (at *p* < 0.05) were employed to test statistical differences among means.

## 3. Results and Discussion

### 3.1. Characterizations of Adsorbents

#### 3.1.1. FTIR Analysis

**Figure 1:**
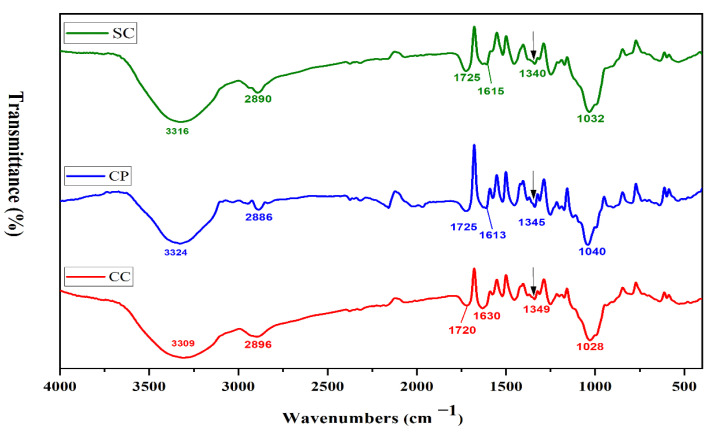
The FTIR spectra of lignocellulosic biomasses, including corn cob (CC), cornstalk piths (CP) and sugarcane bagasse (SC).

FTIR analysis was also employed to confirm the presence of CC, CP and SC biomass components such as lignin, cellulose and hemicellulose. The FTIR spectra of CC, CP and SC biomasses are shown in Figure 1. The presence of cellulose, hemicelluloses and lignin (phenolic+CH_2_OH) was confirmed by the bands 3309–3324 cm^−1^, corresponding to -OH axial deformation and intermolecular hydrogen-bonded H–O–H stretching [51]. Moreover, the deconvolution of broad bands in the 3500–3300 cm^−1^ range was performed to show the differences in hydrogen interactions (Figure S1 and Table S1). The analysis showed that SC biomass had a strong hydrogen bonding interaction (higher intensity), followed by CP biomass, while CC biomass had the lowest density of hydrogen bonding. The fitted peaks centred at 3460–3480 cm^−1^ are assigned to H-bonded -OH group (intermolecular H- bonds) [52]. The characteristic spectral band of lignin assigned to the skeleton vibrations in the phenolic ring (vC=C) at 1517 cm^−1^ was detected [53,54]. The hemicellulose bands at 1173 cm^−1^ referred to acetate groups.

The IR bands at 1609–1630 cm^−1^ related to C=O axial deformation. While the IR band in the region between 1180–950 cm^−1^ presented the fingerprint region of hemicelluloses and cellulose; the bands positioned at 1028–1040 cm^−1^ were assigned to C-O-C skeletal vibration of the polysaccharides ring [51,55]. The total crystalline index (TCI) was calculated (Table 1) from the ratio of peak areas at 1372 cm^−1^ and 2900 cm^−1^ from the FTIR spectrum [56,57]. TCI (A_1372_/A_2900_ cm^−1^) indicates the crystallinity degree of cellulose. The TCI value of CP samples was the lowest among all adsorbents, indicating a higher percentage of amorphous structures in CP. CC samples had a higher TCI ratio than CP and SC, meaning that CC had a more ordered crystalline structure. These findings are matched with the findings of crystallinity behavior collected from XRD results.

#### 3.1.2. Crystallinity Analysis

**Figure 2:**
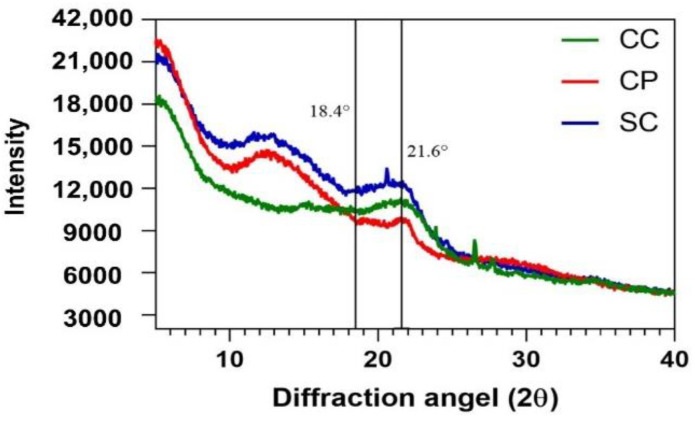
The X-ray diffraction (XRD) spectra of lignocellulosic biomasses, including corn cob (CC), cornstalk piths (CP) and sugarcane bagasse (SC).

Generally, cellulose molecules have both amorphous and crystalline regions, while lignin and hemicellulose only contain amorphous areas. XRD can examine the crystal structure of cellulose and conclude the CrI (%) [54]. Crystallinity is considered the percentage of an ordered crystalline structure in a material. The CrI (%) is related to cellulose, as lignin and hemicellulose only have amorphous structures [58]. XRD analysis of lignocellulosic biomass is challenging because this method measures the crystalline materials’ crystal structure. Notwithstanding, the lignocellulosic material has a complex structure with a high percentage of amorphous structures. Materials with high amorphous contributions exhibit a huge background signal, superimposing the crystalline regions’ clear signals; this phenomenon was observed in our XRD results (Figure 2). As shown in Figure 2, SC and CC biomasses had similar crystalline peaks at 21.5° 2θ, while CP had a wide, non-sharp peak at 21.5°. It was also found that CC had significantly higher CrI (%) compared to SC and CP biomasses (Table 1). These findings were matched with crystallinity properties confirmed by TCI results from FTIR analysis (Table 1). Thus, it could be concluded that the cellulose of CC biomass has more crystalline regions in its structure compared to the cellulose structure of CP and SC.

#### 3.1.3. Microstructure of Adsorbents

**Figure 3:**
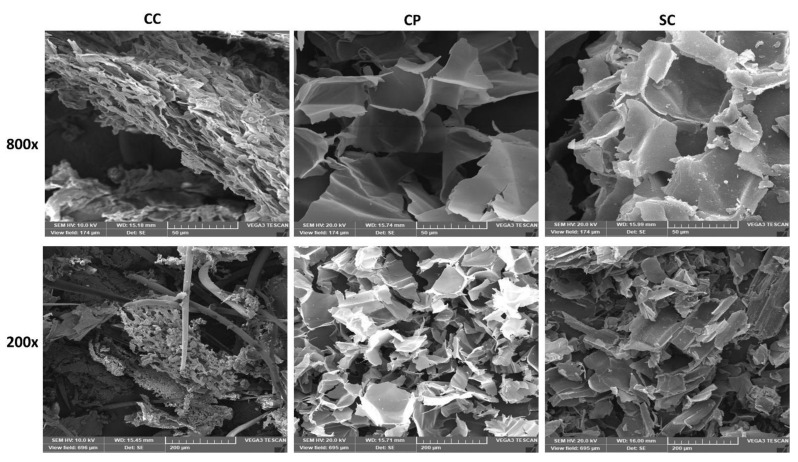
The SEM micrographs of lignocellulosic biomasses; including corn cob (CC), cornstalk piths (CP) and sugarcane bagasse (SC).

The differences in microstructure and element content of the adsorbents were observed using SEM and EDX analysis. The surface morphology and elements content could help in understanding the effects of the lignocellulosic adsorbents on the physicochemical properties of the used edible oils. As shown in Figure 3, CC biomass had an irregular surface and hollow tubular structure with different sizes and shapes of the particles. It was noticed that CC biomass is like wood in terms of mechanical and microstructure properties [59]. Moreover, the microstructure of CP and SC had a more defined structure, and homogenous sponge-like tissues with visible pores were observed in the SEM images of SC (Figure 3). The EDX analysis (Table 2) revealed that CC had higher carbon levels than other adsorbents, while SC and CP biomasses had significantly higher oxygen values compared to CC. Higher oxygen levels could indicate the presence of oxygenated functional groups (e.g., carboxyl, carbonyl, hydroxyl, etc.) that might interact with adsorbates [24].

### 3.2. Acid and Peroxide Values

Acid value (AV) is a widely used parameter to evaluate oil quality; it is a reliable and fast method to check oil acidity during frying. Generally, the AV must be less than 1.0 mg KOH/g oil [36,60]. Table 3 presents the changes in the acidity of the used sunflower oil before and after treatment with lignocellulosic biomasses. The AV of fresh oil was 0.11 mg KOH/g oil and increased after frying for 20 h to 0.63 mg KOH/g oil. The AV increased with the increase of frying hours due to the hydrolysis reaction. The water is released from the potato during frying in the form of vapor, which then interacts with triglycerides, producing di-mono glyceride and free fatty acids [61]. Another reason for the increase in the AV could be the accumulation of secondary oxidation substances [62]. The treatment of fried oils with CC, CP and SC biomasses decreased the AV by 8%, 14% and 19%, respectively, compared to the oil samples fried for 20 h. This could be due to the hydrophilicity nature of lignocellulosic materials, adsorbing vapor from used oil samples and thus slowing the chemical reactions [29,63].

Moreover, the SC biomass showed a more porous structure, as shown in SEM images (Figure 3). This could result in more adsorption positions, a larger specific surface area and a higher binding possibility with free fatty acids [31]. These features could improve the adsorption capacity of these adsorbents and reduce acid values

The peroxide value (PV) is a widely used analytical method to measure hydroperoxides formed at the primary oxidation stage; it was determined by iodometric titration assay [64]. As shown in Table 4, the initial PV of fresh oil was 4.63, then increased to 11.10 after 12 frying hours and thereafter declined to 9.45 meq O_2_/kg after frying for 20 h. The decline in the PV after 12 h of frying could be due to the decomposition of hydroperoxides under the high-temperature condition, forming carbonyl and aldehydic components [65]. The results exhibited the ability of lignocellulosic biomasses to reduce the PV of different oil samples. After applying CC, CP and SC to used oils (20 h), the PVs were reduced to 8.23, 8.96 and 6.91 meq O_2_/kg, respectively. SC biomass adsorbents induced a significant (*p* < 0.05) reduction in PV with lower PVs than CC- and CP-treated oils. This agreed with the results of Ali and El-Anany [36], who found that using 3% sugarcane bagasse ash adsorbent reduced the sunflower oil's PV.

### 3.3. p-AV and TOTOX

PV measures hydroperoxides (initial oxidation products), which decompose to carbonyl components and aldehyde at a longer frying or higher temperature. Thus, PV does not provide a complete evaluation of oil oxidation. Unsaturated aldehydes induce the oils’ off-flavors. *p*-AV determine the secondary oxidation products, especially aldehydes, by color reaction [66,67]. Table 5 displays the *p*-AV of the used oil samples before and after adding SC, CP and CC as natural adsorbents. *p*-AV gradually increased by increasing the frying hours due to the accumulation of secondary oxidation products. *p*-AV of fresh oil was 16.40 and reached 98.45 after 20 h of frying. Using CC, CP and SC adsorbents reduced the *p*-AV of the used oils (20 h) by 9.7, 15.2 and 20.8%, respectively. These findings indicate that lignocellulosic materials have a high affinity with aldehydes and ketones, probably due to the presence of the OH group, which binds with aldehydes and ketones via carbonyl groups [64]. The EDX analysis showed that SC had more oxygen levels than CC and CP, indicating the existence of oxygenated functional groups that could interact with aldehydes, reducing the *p*-AV. Moreover, the FTIR and XRD results showed that the cellulose structure of CC biomass has more crystalline regions than SC and CP. This indicates that more amorphous regions in lignocellulosic biomasses (SC and CP) could improve the absorbance of oxidation residues from fried oils. However, more studies are needed to investigate the relationship between the crystallinity of lignocellulosic materials and the absorbance capacity and oil quality.

The TOTEX value is an indicator of the total oxidation state in oils; it is calculated from PV and *p*-AV [67]. The changes in the TOTOX values are displayed in Table 6. Like *p*-AV, the TOTOX value steadily increased with frying and reached 117.35 after 20 h of frying. Furthermore, the results indicated that the TOTOX value of the used oils (20 h) treated with CC, CP and SC decreased to 105.3, 101.5 and 91.74, respectively. CC biomass was shown the highest TOTOX value, followed by CP, while SC treatment had the lowest TOTOX value. These findings are matched with the PV and *p*-AV results.

### 3.4. Fatty Acid Composition

The fatty acid composition of oil samples is listed in Table 7. The fatty acid profile of oil changed after 20 h of frying, the proportion of unsaturated fatty acids (USFA) decreased to 84.02%, and the proportion of saturated fatty acids (SFA) increased to 15.98%. These changes may be due to the destruction of USFA by polymerization and oxidation of oil at high temperatures [68,69]. The ratio of linoleic acid to palmitic acid indicates the degree of deterioration of frying oils [60,70]. In this study, the ratio of linoleic acid to palmitic acid decreased with increased frying time. Similar findings were reported by Sharoba and Ramadan [70], who found that the ratio of linoleic acid to palmitic acid of palm olein, cottonseed and sunflower oils decreased after increasing the frying time up to 16 h. All adsorbents induced changes in the fatty acid profile of the used oil. In the case of the used oils (20 h), the linoleic acid (C18:2) increased from 58.84% (before treatment) to 61.39%, 61.99% and 61.05% for CC, CP and SC treatments, respectively. Oleic acid decreased in oil treated with CC, CP and SC by 6.99%, 7.11% and 5.21%, respectively. Lignocellulosic adsorbents (CC, CP and SC) reduced the content of USFA in the case of the fried oils for 12 h compared to used oils (12 h). These findings were consistent with the findings of Udomkun et al. [9], who found that cellulose acetate combined with commercial adsorbents reduced the content of USFA.

### 3.5. Color Analysis

Color is another important indicator of frying oil quality. Table 8 shows the effect of adsorption treatments using lignocellulosic biomasses on the oil color. The L* value refers to the brightness from zero value (full dark) to 100 value (full brightness), a* value indicates the level of greenness with a negative and the level of redness with a positive value, while the b* value ranges from blueness as negative and yellowness as a positive value [19]. Compared to fresh oils, after 20 h of potato frying, the color of the used oils tends to be dark (L* = 35.96, a* = 1.78 and b* = 18.60). These changes in the oil color can occur because of the Maillard reaction that occurred between the potato carbohydrates and their amino acids. In addition, the increase in the release of some pigments from frying products. Another reason could be the accumulation of conjugated oxidation products induced by polymerization, oxidation and pyrolysis [19,71]. There was a slight, but not significant, improvement in L* value in SC-treated oil, while other adsorbents were ineffective in recovering the color of the used oils.

## 4. Conclusions

Lignocellulosic biomasses can be considered effective natural adsorbents to increase the quality of edible oils. However, the effects of these adsorbents on the physicochemical properties and quality of fried sunflower oils depended on their type and structure. The XRD analysis of adsorbents showed that CC biomass had the highest crystallinity index (CI, %), followed by SC and CP. In most cases, SC biomass was the most effective adsorbent to improve the used oils’ quality, followed by CP, then CC. SC biomass reduced the acid, peroxide and *p*-AV of fried oils.

Moreover, the lightness of the used oils slightly improved after using SC biomass. The fatty acid analysis showed that using SC and CP biomasses did not significantly alter the fatty acid composition of the used oils. Based on our results, it is recommended to extract cellulose from SC biomass and examine its effect on the physicochemical properties of the used oils. It could also be suggested to study the correlation between the crystallinity degree of lignocellulosic materials and the absorbance capacity and oil quality. Moreover, the infrared spectroscopy technique could be used in future studies to investigate the structural changes of oils.

## Figures and Tables

**Table 1:** The total crystalline index (TCI) from FTIR parameters for crystallinity assessment and the crystallinity index (CrI%) from XRD data of adsorbents; including corn cob (CC), cornstalk piths (CP) and sugarcane bagasse (SC). Means with different letters (^a^, ^b^, ^c^) within each column indicate statistically significant differences between the samples after Duncan’s analysis (*p* < 0.05).

Samples	TCI (A_1372_/A_2900_)	CrI (%)
CC	0.53 ± 0.08 ^a^	53.32 ± 1.13 ^a^
CP	0.40 ± 0.05 ^c^	48.53 ± 0.92 ^b^
SC	0.45 ± 0.06 ^b^	49.24 ± 0.24 ^a,b^

**Table 2:** Element analysis (mass %) of lignocellulosic biomasses, including corn cob (CC), cornstalk piths (CP) and sugarcane bagasse (SC) using Energy Dispersive X-ray spectroscopy (EDX). Means with different letters (^a^, ^b^) within each column indicate statistically significant differences between the samples after Duncan’s analysis (*p* < 0.05). Post-hoc tests were not performed if there were fewer than three groups.

Samples	Carbon	Oxygen	Calcium	Potassium	Cobalt
CC	69.52 ± 3.35 ^a^	27.87 ± 0.75 ^b^	-	2.23 ± 0.95 ^a^	0.59 ± 0.02
CP	57.53 ± 2.63 ^a,b^	35.25 ± 0.92 ^a,b^	4.14 ± 0.13	3.26 ± 0.23 ^a^	-
SC	53.30 ± 1.84 ^b^	39.34 ± 1.22 ^a^	5.67 ± 0.17	1.88 ± 0.27 ^a^	-

**Table 3:** Acid values (mg KOH/g oil) of the used and treated oils with different lignocellulosic biomasses; corn cob (CC), cornstalk piths (CP) and sugarcane bagasse (SC). Means with different letters (^a^, ^b^, ^c^) within each column indicate statistically significant differences between the samples after Duncan’s analysis (*p* < 0.05). The numbers 4, 8, 12, 16 and 20 indicate the frying period by (h, hours).

	0 h	4 h	8 h	12 h	16 h	20 h
Fresh oil	0.11 ± 0.00	-	-	-	-	-
Used oil	-	0.32 ± 0.01 ^a^	0.39 ± 0.05 ^a^	0.44 ± 0.02 ^a^	0.59 ± 0.03 ^a^	0.63 ± 0.02 ^a^
CC-treated oil	-	0.22 ± 0.01 ^b^	0.27 ± 0.00 ^b^	0.37 ± 0.05 ^b^	0.50 ± 0.08 ^a,b^	0.58 ± 0.03 ^a,b^
CP-treated oil	-	0.17 ± 0.01 ^c^	0.28 ± 0.02 ^b^	0.35 ± 0.01 ^b^	0.49 ± 0.04 ^a,b^	0.54 ± 0.00 ^b,c^
SC-treated oil	-	0.14 ± 0.03 ^c^	0.23 ± 0.03 ^b^	0.34 ± 0.02 ^b^	0.41 ± 0.09 ^b^	0.51 ± 0.02 ^c^

**Table 4:** Peroxide values (meq O_2_/kg) of the used and treated oils with different lignocellulosic biomasses; corn cob (CC), cornstalk piths (CP) and sugarcane bagasse (SC). Means with different letters (^a^, ^b^, ^c^) within each column indicate statistically significant differences between the samples after Duncan’s analysis (*p* < 0.05). The numbers 4, 8, 12, 16 and 20 indicate the frying period by (h, hours).

	0 h	4 h	8 h	12 h	16 h	20 h
Fresh oil	4.63 ± 1.03	-	-	-	-	-
Used oil	-	9.11 ± 0.13 ^a^	8.72 ± 2.84 ^a^	11.10 ± 0.89 ^a^	10.96 ± 1.39 ^a^	9.45 ± 0.56 ^a^
CC-treated oil	-	3.43 ± 0.29 ^c^	5.22 ± 0.77 ^b^	9.37 ± 1.45 ^a^	6.21 ± 0.96 ^b^	8.23 ± 1.76 ^a^
CP-treated oil	-	5.26 ± 1.30 ^b^	6.40 ± 0.76 ^a,b^	9.02 ± 0.80 ^a^	4.99 ± 0.93 ^b^	8.96 ± 0.61 ^a^
SC-treated oil	-	2.55 ± 0.41 ^c^	5.19 ± 0.44 ^b^	5.97 ± 1.03 ^b^	5.01 ± 0.92 ^b^	6.91 ± 0.12 ^b^

**Table 5:** *p*-AV of the used and treated oils with different lignocellulosic biomasses; corn cob (CC), cornstalk piths (CP) and sugarcane bagasse (SC). Means with different letters (^a^, ^b^, ^c^, ^d^) within each column indicate statistically significant differences between the samples after Duncan’s analysis (*p* < 0.05). The numbers 4, 8, 12, 16 and 20 indicate the frying period by (h, hours).

	0 h	4 h	8 h	12 h	16 h	20 h
Fresh oil	16.40 ± 1.32	-	-	-	-	-
Used oil	-	43.23 ± 2.30 ^a^	53.81 ± 5.43 ^a^	69.32 ± 4.75 ^a^	73.89 ± 3.68 ^a^	98.45 ± 6.31 ^a^
CC-treated oil	-	36.91 ± 1.42 ^b^	49.71 ± 3.65 ^b^	61.71 ± 6.15 ^c^	71.35 ± 2.43 ^a,b^	88.84 ± 5.74 ^b^
CP-treated oil	-	31.86 ± 1.97 ^c^	51.79 ± 4.59 ^a,b^	66.39 ± 3.35 ^b^	70.59 ± 2.67 ^a,b^	83.56 ± 4.53 ^c^
SC-treated oil	-	32.17 ± 2.23 ^c^	48.99 ± 2.36 ^b^	60.10 ± 2.84 ^c^	69.63 ± 4.21 ^b^	77.92 ± 3.65 ^d^

**Table 6:** Total oxidation value (TOTOX) of the used and treated oils with different lignocellulosic biomasses; corn cob (CC), cornstalk piths (CP) and sugarcane bagasse (SC). Means with different letters (^a^, ^b^, ^c^, ^d^) within each column indicate statistically significant differences between the samples after Duncan’s analysis (*p* < 0.05). The numbers 4, 8, 12, 16 and 20 indicate the frying period by (h, hours).

	0 h	4 h	8 h	12 h	16 h	20 h
Fresh oil	25.66 ± 2.32	-	-	-	-	-
Used oil	-	61.45 ± 5.31 ^a^	71.25 ± 6.25 ^a^	91.52 ± 4.32 ^a^	95.81 ± 3.81 ^a^	117.35 ± 9.15 ^a^
CC-treated oil	-	43.77 ± 3.84 ^b^	60.15 ± 3.34 ^d^	80.45 ± 5.26 ^b^	83.77 ± 4.43 ^b^	105.3 ± 5.32 ^b^
CP-treated oil	-	42.38 ± 4.19 ^b^	64.59 ± 4.65 ^b^	84.43 ± 3.79 ^b^	80.57 ± 3.32 ^b^	101.5 ± 6.62 ^b^
SC-treated oil	-	38.27 ± 2.27 ^c^	59.37 ± 3.43 ^c^	72.04 ± 4.54 ^c^	79.65 ± 2.73 ^b^	91.74 ± 4.76 ^c^

**Table 7:** Fatty acids profile of fresh, fried (for 12 and 20 h), and adsorbents-treated oils with different lignocellulosic biomasses adsorbents, including corn cob (CC), cornstalk piths (CP), and sugarcane bagasse (SC). Means with different letters (^a^, ^b^) within each row indicate statistically significant differences between the samples after Duncan’s analysis (*p* < 0.05).

Fatty Acids (%)	0 h	12 h				20 h			
	Fresh Oil	Used	CC-Treated Oil	CP-Treated Oil	SC-Treated Oil	Used	CC-Treated Oil	CP-Treated Oil	SC-Treated Oil
C16:0	6.84 ± 0.14 ^b^	7.92 ± 0.22 ^b^	13.92 ± 0.36 ^a^	9.83 ± 0.69 ^b^	9.45 ± 0.84 ^b^	8.78 ± 0.54 ^b^	7.98 ± 1.59 ^b^	8.04 ± 0.54 ^b^	8.32 ± 0.73 ^b^
C18:0	3.41 ± 0.11 ^a^	3.75 ± 0.19 ^a^	4.30 ± 0.66 ^a^	4.28 ± 0.43 ^a^	4.49 ± 0.18 ^a^	4.27 ± 0.62 ^a^	3.52 ± 0.97 ^a^	3.58 ± 0.61 ^a^	3.45 ± 0.84 ^a^
C18:1	21.64 ± 0.90 ^a^	23.63 ± 0.37 ^a^	22.93 ± 0.72 ^a^	25.81 ± 1.21 ^a^	24.44 ± 0.95 ^a^	24.72 ± 0.85 ^a^	22.99 ± 1.10 ^a^	22.7 ± 0.92 ^a^	23.43 ± 1.18 ^a^
C18:2	65.30 ± 1.12 ^a^	62.39 ± 1.13 ^a,b^	52.89 ± 2.31 ^b^	56.68 ± 1.23 ^a,b^	57.27 ± 1.52 ^a,b^	58.84 ± 1.18 ^a,b^	61.39 ± 1.14 ^a,b^	61.99 ± 2.25 ^a,b^	61.05 ± 1.93 ^a,b^
SFA (%) **	12.31 ± 0.63 ^b^	13.78 ± 0.82 ^b^	23.07 ± 2.17 ^a^	17.51 ± 0.65 ^a,b^	17.46 ± 0.74 ^a,b^	15.98 ± 0.59 ^b^	15.39 ± 0.89 ^b^	15.31 ± 0.64 ^b^	15.14 ± 0.54 ^b^
USFA (%) ***	87.69 ± 1.89 ^a^	86.22 ± 1.21 ^a^	76.93 ± 2.46 ^a^	82.49 ± 1.13 ^a^	82.53 ± 1.78 ^a^	84.02 ± 2.15 ^a^	84.61 ± 1.76 ^a^	84.69 ± 2.32 ^a^	84.86 ± 1.86 ^a^

** SFA = total saturated fatty acids. *** USFA = total unsaturated fatty acids.

**Table 8:** Color analysis (L*, a*, and b* values) of fresh, used and treated oils with different lignocellulosic biomasses; including corn cob (CC), cornstalk piths (CP), and sugarcane bagasse (SC); 4, 12, and 20 means the frying period by (h, hours). Means with different letters (^a^, ^b^, ^c^, ^d^) within each column indicate statistically significant differences between the samples after Duncan’s analysis (*p* < 0.05).

Frying Time (h)	Samples	L*	a*	b*
0	Fresh oil	40.91 ± 0.32 ^a^	−1.69 ± 0.03 ^c^	7.38 ± 1.21 ^d^
4	Used oil	38.79 ± 0.41 ^a^	−1.74 ± 0.04 ^c^	13.27 ± 2.34 ^c^
	CC-treated oil	39.47 ± 0.65 ^a^	−1.70 ± 0.02 ^c^	13.32 ± 2.12 ^c^
	CP-treated oil	39.29 ± 0.43 ^a^	−1.98 ± 0.02 ^c^	14.57 ± 1.84 ^b,c^
	SC-treated oil	40.30 ± 1.23 ^a^	−1.96 ± 0.03 ^c^	14.37 ± 2.47 ^c^
12	Used oil	36.82 ± 0.95 ^a^	0.49 ± 0.06 ^a,b,c^	17.65 ± 1.59 ^a,b^
	CC-treated oil	36.94 ± 0.56 ^a^	0.78 ± 0.05 ^a,b,c^	19.23 ± 0.12 ^a^
	CP-treated oil	36.68 ± 0.77 ^a^	0.56 ± 0.06 ^a,b,c^	17.68 ± 0.36 ^a,b^
	SC-treated oil	36.62 ± 0.84 ^a^	0.25 ± 0.02 ^b,c^	17.90 ± 0.83 ^a,b^
20	Used oil	35.96 ± 0.92 ^a^	1.78 ± 0.08 ^a,b^	18.60 ± 0.32 ^a^
	CC-treated oil	35.29 ± 0.75 ^a^	3.31 ± 0.21 ^a,b^	17.76 ± 0.89 ^a,b^
	CP-treated oil	35.55 ± 0.54 ^a^	3.71 ± 0.33 ^a^	18.52 ± 1.10 ^a^
	SC-treated oil	38.51 ± 0.42 ^a^	2.38 ± 0.16 ^a,b^	20.84 ± 0.97 ^a^

## Data Availability

The data presented in this study are available on request from the corresponding author.

## Supplementary Materials

The following supporting information can be downloaded at: https://www.mdpi.com/article/10.3390/foods11193149/s1, Figure S1: Fitted peaks of FTIR spectra at 3300–3500 cm^−1^ of lignocellulosic biomasses, including corn cob (CC), cornstalk piths (CP) and sugarcane bagasse (SC); Table S1: Lorentz components of fitted peaks of lignocellulosic biomasses, including corn cob (CC), cornstalk piths (CP) and sugarcane bagasse (SC).
