# Supplementary material for: Lignocellulosic Biomasses from Agricultural Wastes Improved the Quality and Physicochemical Properties of Frying Oils
Source: Foods. 2022 Oct 10;11(19):3149. doi: 10.3390/foods11193149 (PMC9564338; doi:10.3390/foods11193149)
Supplement: Supplementary file 1 [file foods-11-03149-s001.zip › foods-1924612-supplementary.pdf]

foods

MDPI

Article

Lignocellulosic Biomasses from Agricultural Wastes Improved the Quality and Physicochemical Properties of Frying Oils

Eman Ahmed [1,*], Ashraf Zeitoun [1], Gamal Hamad [2], Mohamed A. M. Zeitoun [1], Ahmed Taha [1,3], Sameh A. Korma [4,5] and Tuba Esatbeyoglu [6,*]

[1] Department of Food Science, Faculty of Agriculture (Saba Basha), Alexandria University, Alexandria 21531, Egypt
[2] Department of Food Technology, Arid Lands Cultivation Research Institute (ALCRI), City of Scientific Research and Technological Applications (SRTA City), New Borg El-Arab 21934, Egypt
[3] Department of Functional Materials and Electronics, Center for Physical Sciences and Technology, Saulėtekio al. 3, 10257 Vilnius, Lithuania
[4] Department of Food Science, Faculty of Agriculture, Zagazig University, Zagazig 44519, Egypt
[5] School of Food Science and Engineering, South China University of Technology, Guangzhou 510641, China
[6] Department of Food Development and Food Quality, Institute of Food Science and Human Nutrition, Gottfried Wilhelm Leibniz University Hannover, Am Kleinen Felde 30, 30167 Hannover, Germany
* Correspondence: eman-abdelaziz@alexu.edu.eg (E.A.); esatbeyoglu@lw.uni-hannover.de (T.E.); Tel.: +20-1212484089 (E.A.); +49-5117625589 (T.E.)

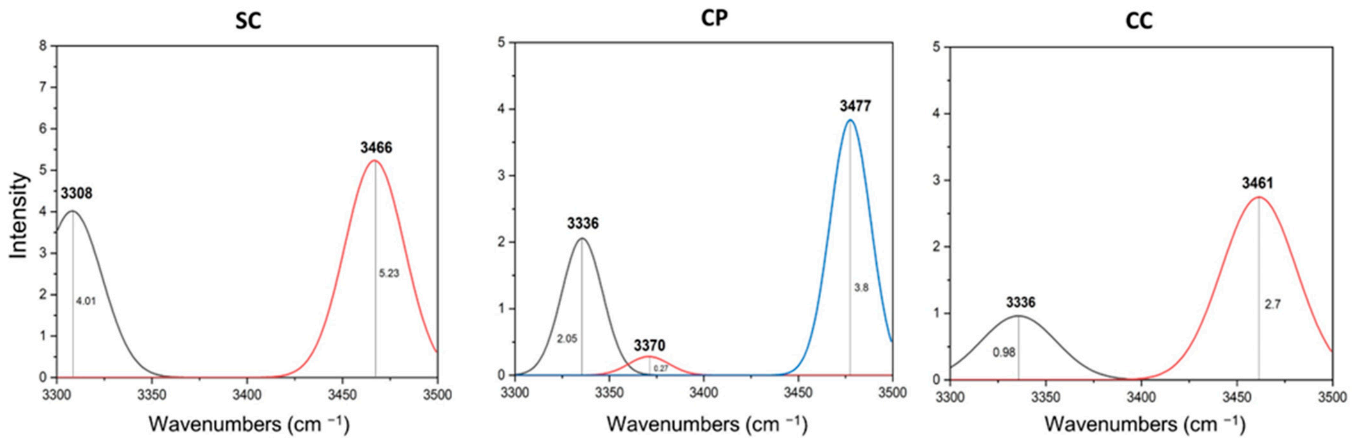

Figure S1. Fitted peaks of FTIR spectra at 3300 - 3500 cm $^{-1}$ of lignocellulosic biomasses, including corn cob (CC), cornstalk piths (CP) and sugarcane bagasse (SC).

Table S1. Lorentz components of fitted peaks of lignocellulosic biomasses, including corn cob (CC), cornstalk piths (CP) and sugarcane bagasse (SC).

	**Peak 1**			**Peak 2**		
	**SC**	**CC**	**CP**	**SC**	**CC**	**CP**
Center (cm $^{-1}$)	3466	3461	3477	3308	3336	3336
Hight (intensity)	5.23	2.70	3.84	4.01	0.98	2.05
FWHM	36.85	46.23	25.32	36.85	46.23	25.31
Area	205.46	135.18	103.40	157.58	47.31	55.48

FWHM, Full width half maximum.
